# Effect of supplementation and withdrawal of selenium-enriched kale sprouts on productivity and egg selenium concentration of laying hens

**DOI:** 10.5713/ab.22.0067

**Published:** 2022-09-07

**Authors:** Anut Chantiratikul, Pinyada Thongpitak, Orawan Arunsangseesod, Eakapol Wangkahart, Kwanyuen Leamsamrong, Worapol Aengwanich, Juan Boo Liang, Wu Xin, Piyanete Chantiratikul

**Affiliations:** 1 Applied Animal and Aquatic Sciences Research Unit and Department of Agricultural Technology, Faculty of Technology, Mahasarakham University, Kantharawichai, Maha Sarakham 44150, Thailand; 2 Department of Chemistry, Faculty of Science and Technology, Rajabhat Maha Sarakham University, Mueang, Maha Sarakham 44000, Thailand; 3 Faculty of Veterinary Sciences, Mahasarakham University, Maha Sarakham 44000, Thailand; 4 Institute of Tropical Agriculture and Food Security, Universiti Putra Malaysia, 43400 Selangor, Malaysia; 5 CAS Key Laboratory of Agro-Ecological Processes in Subtropical Region, Institute of Subtropical Agriculture, Chinese Academy of Sciences, Changsha, Hunan 410125, China; 6 Department of Chemistry, Faculty of Science, Mahasarakham University, Kantharawichai, Maha Sarakham 44150, Thailand

**Keywords:** Egg Selenium Content, Organic Selenium, Selenium-enriched Plant, Selenium Source, Selenium-enriched Yeast

## Abstract

**Objective:**

The aim of this trial was to investigate the effect of supplementation and withdrawal of selenium-enriched kale sprouts (SeKS) on productivity and egg Se concentration of laying hens. Selenium from commercial Se-enriched yeast (SeY) was used as a comparative Se source.

**Methods:**

One-hundred and eighty 61-week-old laying hens were randomly divided into 5 treatment groups with 4 replicates (9 hens each) in a 2×2+1 Augmented Factorial Experiment in a completely randomized design. The experimental diets were basal diet, basal diet supplemented with 0.2 and 0.4 mg Se/kg from SeKS and SeY, respectively. The 8-week feeding trial was divided into 2 periods, namely the Se supplemental period (week 1 to 4) and the Se withdrawal period (week 5 to 8).

**Results:**

Productive performance, egg quality and egg Se concentration of laying hens were not affected by sources of Se (SeKS and SeY) during both, the Se supplemental and withdrawal periods. Egg production and egg Se concentration increased (p<0.05) with increasing levels of Se supplementation. The egg Se concentration increased and reached a peak 1 week after Se supplementation. However, concentration of Se in eggs of hens fed Se from both sources decreased rapidly from the second week of the Se withdrawal period to reach the same egg Se concentration of hens fed the basal diet by the fourth week of the Se withdrawal period.

**Conclusion:**

The efficacy of Se from SeKS on productivity and egg Se concentration in laying hens was comparable to commercial SeY. Thus, SeKS can provide an alternate organic Se source for production of Se-enriched eggs.

## INTRODUCTION

Selenium (Se) is recognized as one of the important essential micronutrients for humans and farm animals, including poultry. Selenium incorporation into proteins involves translational decoding as a component of selenocysteine, the 21st amino acid, used for selenoproteins synthesis [1]. The selenoprotein family consists of 25 eukaryotic genes, which contain Se in its active site. Most selenoproteins, especially glutathione peroxidase (GSH-Px), display antioxidant activities [2]. Poultry researchers have evaluated the performances of laying hens in response to dietary inorganic and organic Se supplementations. The supplementation of organic Se from Se-enriched yeast (SeY) or selenomethionine (SeMet) markedly improved performance, GSH-Px activity and Se concentrations in tissues and eggs of laying hens compared to inorganic Se from sodium selenite (SS) [3–6]. Commercially produced SeY has been widely used as a Se source to produce Se-enriched eggs in more than 25 countries, providing an effective source of Se for humans [7].

Several Se-enriched plants such as mung bean (*Vigna radiata*) [8], malt (*Hordeum vulgare*) [9] and sunflower (*Helianthus annuus*) sprouts [10] have been cultivated as alternative Se sources for humans and farm animals. These studies reported total Se concentration but not Se species in the Se-enriched plants. Hence, the efficacy of Se in different Se-enriched plants requires evaluation. Recently, Se-enriched kale sprouts (SeKS) have been hydroponically produced and studied. A Se speciation study indicated that SeKS predominantly consisted of organic Se in the form of SeMet [11]. Evaluation of dietary SeKS supplementation in broilers, quails and laying hens found that its effectiveness was comparable to SeY [12–14], while enhancing the antioxidant capacities using high doses (0.54 to 2.16 mg Se/kg BW) of SeKS did not result in any toxicological signs and mortality in rats [15]. Similarly, dietary supplementation of Se sourced from SeKS at up to 5 to 10 mg/kg did not cause any toxic effect in laying hens [16]. SeKS extract also exhibited cytotoxicity on Caco-2, MCF-7 and HepG2 human cancer cells [17], suggesting SeKS as a promising Se source for farm animals and humans.

Dynamic changes of Se concentration in eggs after dietary supplementation and withdrawal provide useful information for the production of Se-enriched eggs. Egg Se concentration generally increased and reached the zenith between 1 to 2 weeks post supplementation in both organic and inorganic forms in laying hens [9,18,19]. However, earlier studies indicated that Se concentration in eggs of inorganic Se supplemented hens declined at different rates (1 to 4 weeks) to reach the same level as eggs from non-supplemented hens after dietary Se withdrawal [18,20]. Thus, dynamic changes in egg Se concentration after supplementation and withdrawal of organic Se, particularly for SeKS in laying hens, requires further investigation to better understand its efficacy as an organic Se supplementation source.

This study determined the effect of supplementation and withdrawal of SeKS on productive performance, egg quality, Se retention rate and egg Se concentration in laying hens. Commercial SeY was used as a comparative organic Se source.

## MATERIALS AND METHODS

### Ethics statement of animal care

All experimental procedures were approved by the Institutional Animal Care and Use Committee, Mahasarakham University (Approval No. IACUC-MSU-002/2019).

### Production of Se-enriched kale sprouts

Se-enriched kale sprouts were prepared following the procedure of Maneetong et al [11]. Briefly, kale seeds (*Brassica oleracea var alboglabra* L.) were soaked in water for 15 h, planted into wet sponges and placed in plastic pots, which were fully covered for 3 days for the seeds to germinate. The germinated kale seedlings were exposed to artificial light using a fluorescent lamp and watered with tap water for 4 days. The seedlings were then cultivated in Hoagland’s solution containing 30 mg Se from SS/L for 15 days. The SeKS were then harvested, washed thoroughly with deionized water, dried in a hot air oven (60°C), ground and stored at 4°C until used.

### Birds and experimental procedures

One hundred and eighty 61-week-old Hy-Line Brown laying hens were raised in a poultry house with an evaporative cooling system. The internal temperature was automatically controlled at 24°C with lights on continuously. The hens were randomly divided into 5 treatment groups in a 2×2+1 Augmented Factorial Experiment in a completely randomized design (CRD). Each treatment group consisted of 4 replicates with 9 hens each. Hens in each replicate were placed in wire cages. The basal diet (Table 1) was formulated to meet the nutrient requirements of laying hens [21] with no Se supplementation. Dietary treatments included basal diet, basal diet supplemented with 0.2 and 0.4 mgSe/kg from SeKS, and basal diet supplemented with 0.2 and 0.4 mgSe/kg from SeY (Cytoplex-Se 2000; Phytobiotics Feed Additive Gmbh, Eltville, Germany). The SeKS and SeY were thoroughly mixed separately in the corn and then added to the diet to achieve the assigned Se treatment levels. The experiment was divided into 2 periods: the Se supplemental period (week 1 to 4) when the hens were fed the assigned dietary treatments and the Se withdrawal period (week 5 to 8) when hens were fed the basal diet without Se supplementation. Clean drinking water was available throughout the experimental period.

### Data and sample collections

The diets were randomly sampled at the beginning of the Se supplemental and withdrawal periods, dried and ground for proximate analysis and determined for Se concentration. Initial and final body weights (BW) of hens in each replicate were measured at the beginning and the end of the trial for evaluation of BW changes. Feed intake and egg production of hens in each replicate were recorded daily. Feed conversion ratio (FCR) as feed intake (g) per egg mass (g) was also calculated.

Three eggs per replicate (12 in total) in each treatment were randomly sampled on day 1, 3, 5, 7, and 9 and at the end of week 2 to 8 and stored at 4°C until processed. Six eggs from each sampling treatment were used to determine egg weight, Haugh units, yolk color and eggshell thickness after each collection. Haugh units were estimated by measuring albumen height with an albumen height gauge (TSS-QCD Instruments, York, England). Eggshell thickness and yolk color were measured with a micrometer (395-541-30 BMD-25DM; Mitutoya, Japan) and a yolk color fan (Roche yolk color fan 1993-HMB 50515, Switzerland), respectively. The remaining six eggs were used for Se concentration determination. The first three eggs were cracked and the content thoroughly mixed. Egg albumen and yolk of the remaining three eggs were separated and homogenized. Then, samples of the whole egg, egg albumen and yolk were dried at 65°C for 12 h, ground, pooled by replicate in each treatment, and stored.

Excreta outputs of hens in each replicate were collected in a tray located underneath the cages for two consecutive days at the end of week 2 of the supplementation period, according to the previously proposed procedure [22,23]. The excreta were weighed, kept at 60°C in a drying oven for 24 h or until dry, ground and stored for Se retention rate study.

### Chemical determination and calculation

Nutrient composition of the diets was analyzed [24] for dry matter (DM) (Method 934.01), crude protein (CP) (Method 976.05), ether extract (EE) (Method 920.39), crude fiber (Method 978.10) and ash (Method 942.05). Dried samples of SeKS, SeY, diets, whole egg, egg albumen, egg yolk and excreta were weighed and placed separately into a vessel. Then, 3.0 mL of 1:1 nitric acid and deionized water were added. Samples were digested at 100°C in the digestion block until the solution turned clear. After cooling, 5 mL of HCl was added to the vessel. The samples were again heated to 100°C for 10 min and cooled. Finally, deionized water was poured into the volumetric flask to make up the reduced volume of the digest. The concentration of Se in the samples was analyzed using a Hydride Generation-Atomic Absorption Spectrometer (HG-AAS model VGA-77; Agilent Technologies, Inc., Santa Clara, CA, USA) at maximum wavelength 196.0 nm, quartz cell temperature 850°C and carrier solutions HCl 4 M and NaBH_4_ 0.2% w/v.

The retention rate of Se was calculated as the following equation [22]:


$$\text{Se retention rate }(\%)=[({\text{Se}}_{\text{I}}-{\text{Se}}_{\text{E}})\times 100]/{\text{Se}}_{\text{I}}$$

where Se_I_ is the Se intake and Se_E_ is the Se excreted in manure.

### Statistical analysis

All data of productive performances and Se concentrations in eggs were analyzed using general linear model procedures appropriate for 2×2+1 Augmented Factorial Experiments in a CRD [25]. Treatment differences were determined by orthogonal contrasts i) basal diet vs Se supplemental diets; ii) SeKS vs SeY and iii) levels of Se supplementation. The probability level was considered to be statistically significant at p<0.05.

## RESULTS

Feed ingredients and analyzed chemical composition of the basal diet are presented in Table 1. Analyzed concentrations of Se in the treatment diets are shown in Table 2. Selenium concentrations in SeKS and SeY were 268.12 and 2,266.83 mg/kg DM, respectively. Basal diet and basal diets supplemented with 0.2 and 0.4 mg Se/kg from SeKS and SeY contained 0.10, 0.23, 0.47, 0.24, and 0.46 mg Se/kg DM, respectively.

Sources and levels of Se supplementations did not affect the BW of laying hens. Feed intake and FCR of laying hens in Se supplemental and withdrawal periods were not affected by Se sources and levels (Table 3).

The effects of Se supplementation and withdrawal from different sources on production performance, egg production and quality of the experimental birds were evaluated. Results showed that egg production and egg quality of laying hens fed Se supplemental diets were not different from those fed the basal diet in the Se supplemental and withdrawal periods (Table 3). Sources and levels of Se did not influence egg weight, Haugh unit, egg yolk color and eggshell thickness in both periods. Irrespective of sources, egg production was lower (p = 0.04) for hens supplemented with 0.2 mg Se/kg as compared to 0.4 mg Se/kg during the Se supplemental period; however, this was not observed during the withdrawal period (Table 3).

Irrespective of the sources, Se retention rate of laying hens fed Se supplemental diets was higher (p = 0.002) than for laying hens fed the basal diet. However, sources and levels of dietary Se supplementation had no effect on Se retention rate in laying hens (Table 4).

Egg yolk, egg albumen and whole egg Se concentrations of laying hens fed Se supplemental diets were higher (p = 0.001) than for laying hens fed the basal diet in both supplemental and withdrawal periods. During the supplemental period, no differences were recorded in Se concentrations in egg yolk, egg albumen and whole egg between the two sources of Se. Egg yolk, egg albumen and whole egg Se concentrations were enhanced (p<0.05) with increasing levels of Se supplementation. However, these enhancements were not observed in the Se withdrawal period (Table 5).

**Figure 1:**
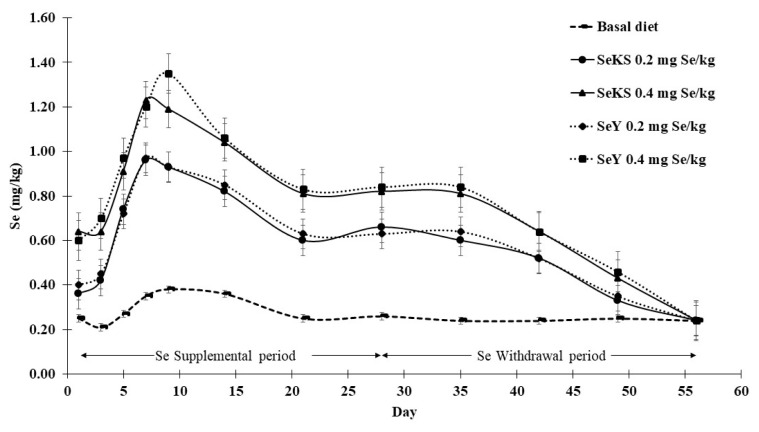
Dynamic changes of whole egg selenium concentration of laying hens fed basal diet and selenium supplemental diets during the supplemental (day 1 to 28) and withdrawal (day 29 to 56) periods. SeKS, Se-enriched kale sprouts; SeY, Se-enriched yeast.

Se concentration in whole egg increased rapidly from day 1 and peaked at day 7 to 9 after receiving Se supplemental diets, thereafter decreasing and stabilizing after 2 weeks. Source of Se did not affect the rate of Se concentration deposition in whole eggs but whole egg Se concentration increased with increasing levels of Se supplementation from 0.2 to 0.4 mg/kg (Figure 1).

During the withdrawal period (from day 29 to 56) laying hens in all experimental groups received the same basal diet. Results showed that whole egg Se concentration remained near stable for a week (day 29 to 35) after Se withdrawal but decreased substantially thereafter to reach a similar level to the basal diet at the end (day 56) of the experiment. Although declining during the withdrawal period, levels of whole egg Se concentration in the 0.4 mg Se/kg groups were higher at all times than in the 0.2 mg Se/kg groups until day 49 (Figure 1).

## DISCUSSION

Previous studies reported that dietary supplementation of SeY or SeMet ranging from 0.1 to 3.0 mg/kg did not affect productive performance and egg quality of laying hens [6,26–28], while dietary supplementation of SeKS up to 0.3 mg/kg did not alter feed intake, FCR, egg production and egg quality of laying hens [14]. Our results during the Se supplemental period concurred with the above reports. However, the present result showed that egg production increased with increasing levels of Se supplementation. Some studies reported positive responses of egg production in laying hens receiving higher dosage of both inorganic and organic Se [4,5,29,30], while others found that incremental levels of Se supplementation did not influence the laying performance [23,31]. Inconsistent egg productivity in response to Se dosage requires further investigation. One contributing factor that enhanced egg production in this study was increased GSH-Px activity [3,16,32], resulting in higher antioxidant capacity as an indirect positive effect on egg production of laying hens.

Productivity and egg quality of laying hens were not affected by the withdrawal of dietary Se supplementation. Hens in all treatments were fed a basal diet containing 0.1 mg Se/kg during the 4-week Se withdrawal period. This met the recommended dietary Se requirement of 0.15 mg/kg for laying hens [21]. Hence, hens in all the treatment groups were fed with sufficient Se during the withdrawal period and productive performance was not affected. Se concentration in basal diet has been highlighted. Previous studies in laying hens, broilers and quails also found that Se concentration in the basal diets met or slightly exceeded their respective requirements, ranging from 0.11 to 0.25 mg/kg [12,13,16,33], implying that conventional feed ingredients commonly used in poultry diets in Thailand and other countries in the region contain adequate Se for egg production. However, no data on total Se concentration and Se species in animal feedstuffs is available to confirm this speculation. Sirichakwal et al [34] reported a large variation in the Se content of various food ingredients, especially foods of Thai plant origin. They hypothesized that huge variations in Se soil concentrations, ranging from 14 to 129 μg/kg were a likely factor causing variations in Se levels of the studied plants. Therefore, more scientific data on Se concentration in animal feed ingredients are needed for recommendations of Se supplementation in animal diets.

The efficacy and retention rate of Se depend on chemical forms in the Se sources. Organic Se, especially SeMet, has a higher retention rate than inorganic Se. SeY is a commercial organic Se source for animal production worldwide, containing organic Se mostly in the form of SeMet, varying from 50% to 70% [35]. We recently reported that hydroponically grown SeKS contained up to 42% SeMet [11]. The organic Se forms in SeKS and SeMet resulted in higher Se retention rate in hens fed Se supplemental diets compared to hens fed diets without Se supplement. Latshaw and Osman [36] also reported greater Se retention in hens fed SeMet compared to hens fed diets with no Se supplement. One of the objectives of this trial was to compare the retention rate of Se from SeKS with commercial SeY for Se-enriched egg production. During the Se supplemental period, no differences in Se retention rate between the two organic Se sources were found, leading to no differences in concentrations of Se in egg yolk, egg albumen and whole egg. Our results concurred with previous studies [5,6,16,28] that Se concentration in eggs increased with increasing levels of Se supplementation from SeY and SeKS, indicating that SeKS can serve as an alternative organic Se source to commercial SeY for enhancement of Se concentration in eggs. Furthermore, our results showed that Se concentrations in egg yolk, egg albumen and whole egg of hens fed Se supplemental diets at 0.2 and 0.4 mg/kg were 2.34 to 2.60-fold and 3.13 to 3.37-fold higher than hens fed the basal diet, respectively. SeMet can be metabolically incorporated non-specifically into protein by randomly replacing methionine [2,37], resulting in higher Se concentration in eggs with increasing levels of Se supplementation from SeKS and SeY.

Laying hens in all experimental groups were fed the same basal diet during the 4-week withdrawal period, and average Se concentrations of egg yolk, egg albumen and whole egg of hens previously fed Se supplemental diets remained higher than hens fed the basal diet. Both dietary Se and stored Se in the form of SeMet can be metabolically utilized for selenoprotein synthesis [2]. However, it is scientifically assumed that when the hen receives adequate Se, the ingested Se is the main Se source used for selenoprotein synthesis instead of the stored Se in body protein that is used when inadequate Se occurs. Hence, the average Se concentration in eggs of hens in the Se supplemental groups remained higher than hens in the basal diet group during the Se withdrawal period.

Dynamic changes in egg Se concentrations after dietary Se supplementation in laying hens have been extensively studied. Payne et al [38] and Chantiratikul et al [19] reported that egg Se concentration mirrored the level of organic Se in the diet and reached a plateau from day 4 to 7 post-feeding. In our study, highest egg Se concentration was recorded from day 7 to 9 during the Se supplemental period. However, others reported a longer duration (12 to 28 days) to reach maximal egg Se concentrations in hens fed inorganic or organic Se [6,9,18,23]. These inconsistent results suggest that further elucidation of egg selenium content is required.

Research data regarding the effect of withdrawal of dietary Se from hens supplemented on egg Se concentration is scant. Arnold et al [18] observed that withdrawal of dietary inorganic Se in the form of SS (8 mg Se/kg) reduced egg Se concentration abruptly, and concentration returned to normal within about 8 days, while Ort and Latshaw [20] reported a longer period of 4 weeks after withdrawal of SS supplementation (5 to 9 mg Se/kg). No reports on the dynamic changes of egg Se concentration after withdrawal of organic Se supplementation are available. This study compared Se supplementation withdrawal between organic Se from SeKS and SeY. Our results showed that egg Se concentration of hens fed Se from both sources reduced continuously to the same level as hens fed the basal diet after 4 weeks of withdrawal. Our findings concurred with the rate of decline of inorganic Se from SS previously reported [20], and also showed that the rate of decline of Se concentration in eggs for hens fed higher (0.4 mg Se/kg) Se supplement was greater than for hens fed lower (0.2 mg Se/kg), with both achieving the same level at 4 weeks after withdrawal. Based on this data, eggs should not be sold as Se-enriched after Se supplementation has been withdrawn for 1 week when SeY and SeKS are used as Se sources.

In conclusion, the effects of dietary supplementation of SeKS and SeY and their withdrawal resulted in similar productivity, Se retention rate and egg Se concentration in laying hens. SeKS could serve as an alternative organic source for production of Se-enriched eggs.

## Figures and Tables

**Table 1:** Feed ingredients and chemical composition of the basal diet^1)^

Items	% DM
Ingredient	
Corn	56.00
Extruded soybean	9.70
Rice bran	6.25
Soybean meal (44% CP)	18.00
Soybean oil	3.00
Dicalcium phosphate	2.50
Oyster shell meal	4.50
DL-methionine	0.10
L-lysine	0.10
Salt	1.25
Vitamin-mineral premix^2)^	0.25
Chemical composition	
Dry matter	91.96
Crude protein	16.62
Ether extract	6.70
Crude fiber	2.72
Ash	11.68
Lysine^3)^	0.89
Methionine+cysteine^3)^	0.74
Ca^3)^	4.15
Available P^3)^	0.38
ME^3)^ (kcal/kg)	2,950

DM, dry matter; CP, crude protein; ME, metabolizable energy.

1) SeKS and SeY were mixed in corn and added to the diet to achieve the treatment levels.

2) Vitamin-mineral premix (per kg): Vitamin A 4,000 IU, Vitamin D_3_, 800 IU, Vitamin E 4.8 IU, Vitamin K 30.60 mg, Vitamin B_1_ 0.60 mg, Vitamin B_2_ 1.60 mg, Vitamin B_6_ 1.60 mg, Vitamin B_10_ 0.007 mg, Pantothenic acid 4.00 mg, Niacin 8.80 mg, Folic acid 0.17 mg, Biotin 0.07 mg, Folic 24 mg, Manganese 28 mg, Zinc 20 mg, Copper 3.2 mg, Cobalt 0.20 mg, and Iodine 0.28 mg.

3) Calculated value.

**Table 2:** Selenium concentrations in SeKS, SeY, and experimental diets

Item	Se (mg/kg DM)
SeKS	268.12
SeY	2,266.83
Basal diet	0.10
Basal diet plus 0.2 mg Se/kg from SeKS	0.23
Basal diet plus 0.4 mg Se/kg from SeKS	0.47
Basal diet plus 0.2 mg Se/kg from SeY	0.24
Basal diet plus 0.4 mg Se/kg from SeY	0.46

SeKS, Se-enriched kale sprouts; SeY, Se-enriched yeast; DM, dry matter.

**Table 3:** Effect of supplementation and withdrawal of selenium sources on performance, egg production and egg quality of laying hens

Item	Basal diet	SeKS (mg Se/kg)		SeY (mg Se/kg)		SEM	p-value^1)^			
		
		0.2	0.4	0.2	0.4		B	S	L	S×L
Initial weight (kg)	1.97	1.95	1.90	1.90	1.94	0.02	-	-	-	-
Final weight (kg)	2.04	2.02	1.98	1.97	2.10	0.02	0.29	0.82	0.92	0.38
BW changes (kg)	0.07	0.07	0.08	0.07	0.06	0.003	0.85	0.33	0.89	0.69
	-------------------- Se supplemental period (wk 1 to 4) --------------------									
Feed intake (g/d)	102.58	103.65	102.82	101.94	102.42	0.62	0.94	0.50	0.91	0.44
FCR (g/g)	2.10	2.12	2.10	2.25	2.12	0.03	0.46	0.22	0.19	0.11
Egg production (%)	77.97	75.19	77.51	70.21	72.51	1.24	0.27	0.25	0.04	0.10
Egg weight (g)	63.27	63.67	63.83	63.94	64.20	0.49	0.67	0.81	0.87	0.88
Haugh units (HU)	76.50	77.62	79.31	83.43	82.68	1.19	0.16	0.09	0.85	0.13
Yolk color^2)^	8.03	8.12	8.09	8.15	7.97	0.06	0.77	0.77	0.50	0.89
Shell thickness (mm)	0.28	0.28	0.28	0.27	0.28	0.03	0.61	0.32	0.32	0.17
	-------------------- Se withdrawal period (wk 5 to 8) --------------------									
Feed intake (g/d)	100.75	102.41	101.01	100.45	101.17	0.67	0.78	0.59	0.84	0.41
FCR (g/g)	2.12	2.09	2.08	2.18	2.15	0.02	0.91	0.17	0.75	0.21
Egg production (%)	76.52	78.80	77.07	75.03	73.21	1.33	0.89	0.29	0.62	0.39
Egg weight (g)	62.53	63.48	62.56	62.50	61.32	0.47	0.96	0.34	0.36	0.55
Haugh units (HU)	83.25	81.56	82.56	81.50	79.81	1.19	0.57	0.64	0.91	0.99
Egg yolk color^2)^	7.69	7.75	7.87	7.56	7.75	0.08	0.97	0.25	0.70	0.51
Shell thickness (mm)	0.25	0.23	0.24	0.25	0.24	0.03	0.58	0.23	0.99	0.10

SeKS, Se-enriched kale sprouts; SeY, Se-enriched yeast; SEM, standard error of the mean; BW, body weight; FCR, feed conversion ratio (feed intake/egg mass).

1) B, basal diet vs others; S, SeKS vs SeY; L, levels of Se supplementation; S×L, Se sources×levels.

2) Egg yolk color, level of egg yolk color intensity score from yellow (1 point) to orange (15 points).

**Table 4:** Selenium retention rate of laying hens fed the basal diet or diets supplemented with additional selenium

Item	Basal diet	SeKS (mg Se/kg)		SeY (mg Se/kg)		SEM	p-value^1)^			
		
		0.2	0.4	0.2	0.4		B	S	L	S×L
Se intake (mg DM/d)	0.010	0.023	0.048	0.024	0.048	0.003	0.001	0.84	0.001	0.57
Se output (mg DM/d)	0.003	0.005	0.010	0.005	0.010	0.001	0.001	0.99	0.001	0.99
Se retention rate (%)	70.63	76.39	78.24	75.92	78.55	0.81	0.002	0.94	0.080	0.78

SeKS, Se-enriched kale sprouts; SeY, Se-enriched yeast; SEM, standard error of the mean; DM, dry matter.

1) B, basal diet vs others; S, SeKS vs SeY; L, levels of Se supplementation; S×L, Se sources×levels.

**Table 5:** Selenium concentrations (mg/kg DM) in egg yolk, egg albumen and whole egg of laying hens in the selenium supplemental and withdrawal periods

Item	Basal diet	SeKS (mg Se/kg)		SeY (mg Se/kg)		SEM	p-value^1)^			
		
		0.2	0.4	0.2	0.4		B	S	L	S×L
	-------------------- Se supplemental period (wk 1 to 4) --------------------									
Egg yolk	0.32	0.75	1.00	0.78	1.07	0.08	0.001	0.06	0.001	0.50
Egg albumen	0.30	0.78	0.97	0.77	1.01	0.08	0.001	0.54	0.006	0.91
Whole egg	0.31	0.76	0.98	0.77	0.98	0.08	0.001	0.79	0.008	0.79
	-------------------- Se withdrawal period (wk 5 to 8) --------------------									
Egg yolk	0.24	0.42	0.53	0.44	0.55	0.03	0.001	0.26	0.08	0.65
Egg albumen	0.24	0.43	0.51	0.44	0.54	0.03	0.001	0.07	0.07	0.08
Whole egg	0.24	0.42	0.53	0.44	0.55	0.03	0.001	0.08	0.07	0.11

SeKS, Se-enriched kale sprouts; SeY, Se-enriched yeast; SEM, SEM, standard error of the mean; DM, dry matter.

1) B, basal diet vs others; S, SeKS vs SeY; L, levels of Se supplementation; S×L, Se sources×levels.
